# Supplementary figures and images for: Anti-Tumor Effect of Turandot Proteins Induced via the JAK/STAT Pathway in the mxc Hematopoietic Tumor Mutant in Drosophila
Source: Cells. 2023 Aug 11;12(16):2047. doi: 10.3390/cells12162047 (PMC10453024; doi:10.3390/cells12162047)

Figure S1

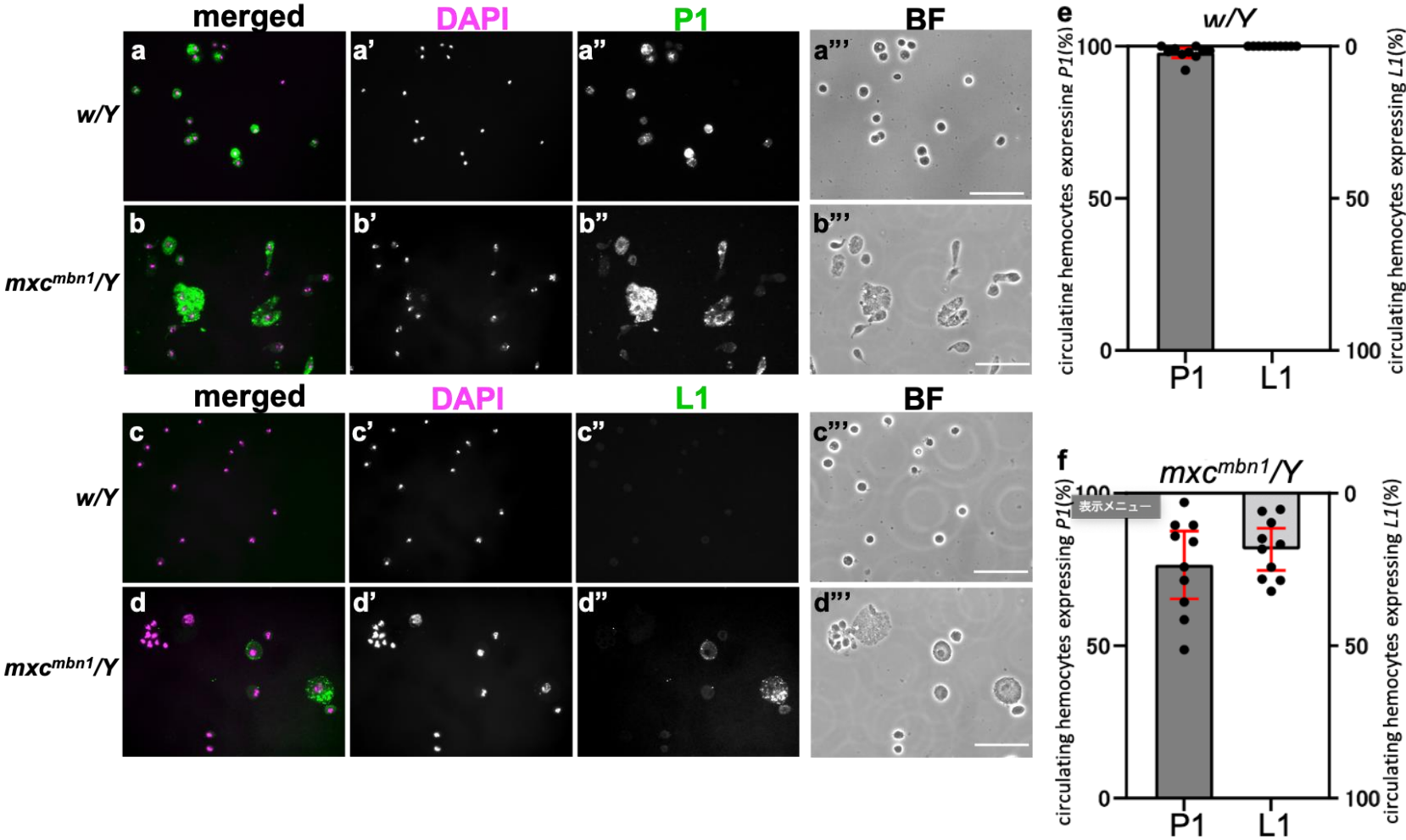

**Figure S2**

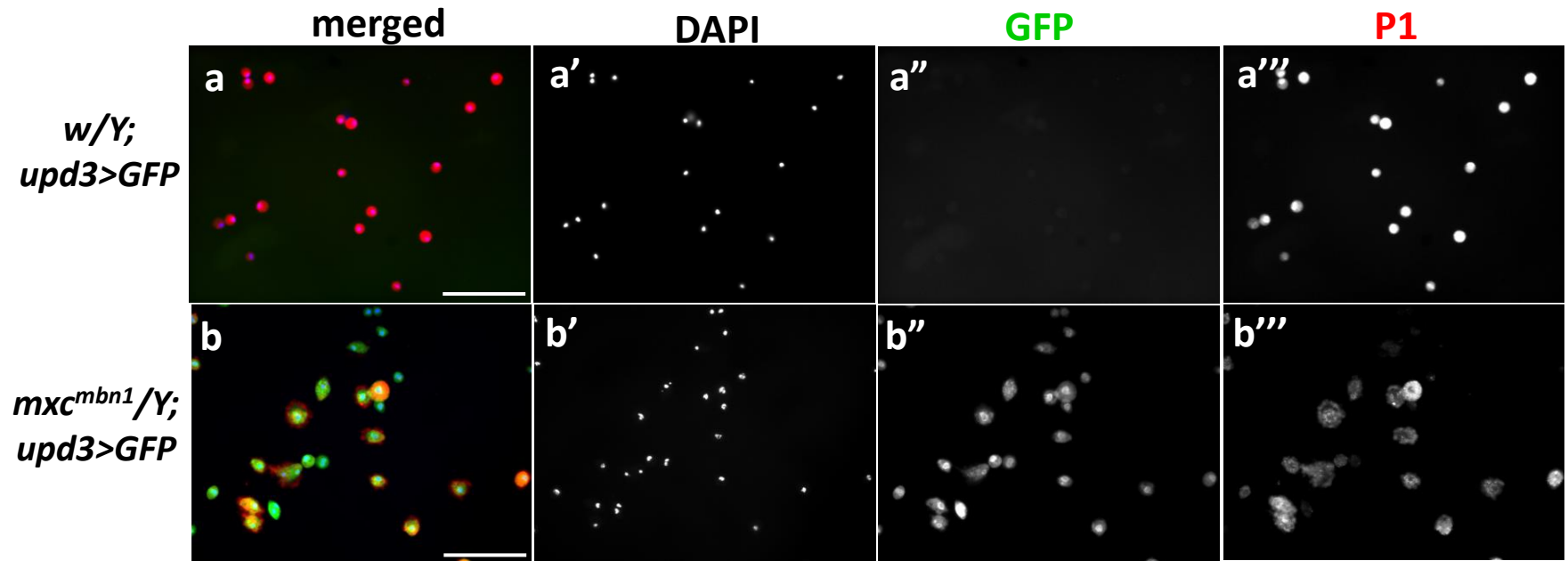

Figure S3

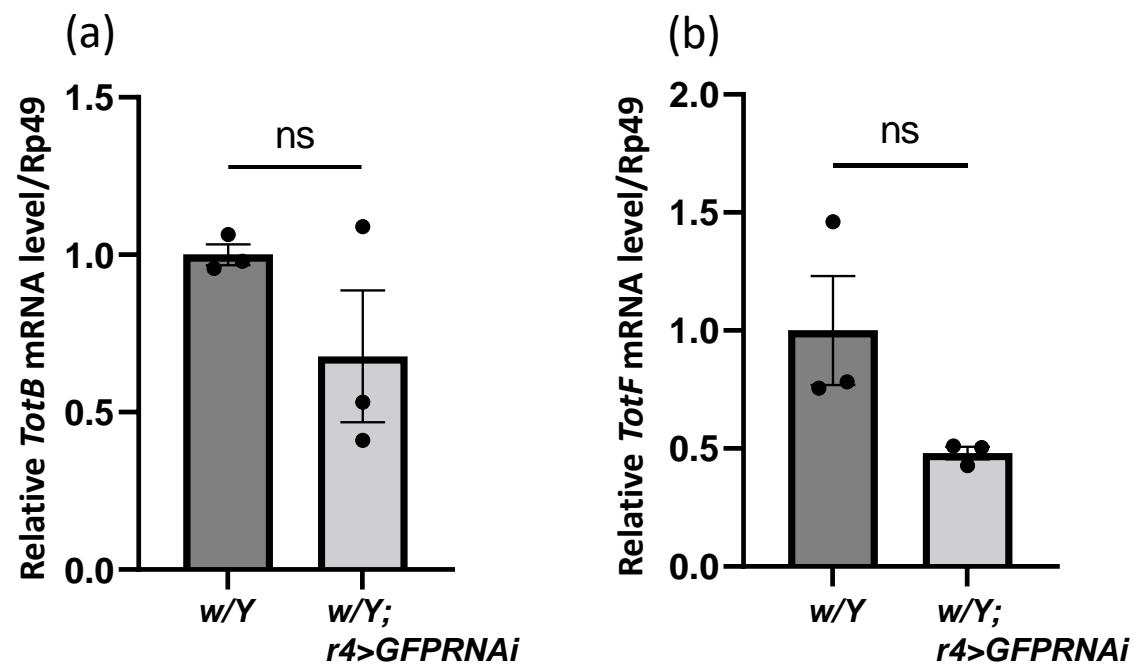

Figure S4

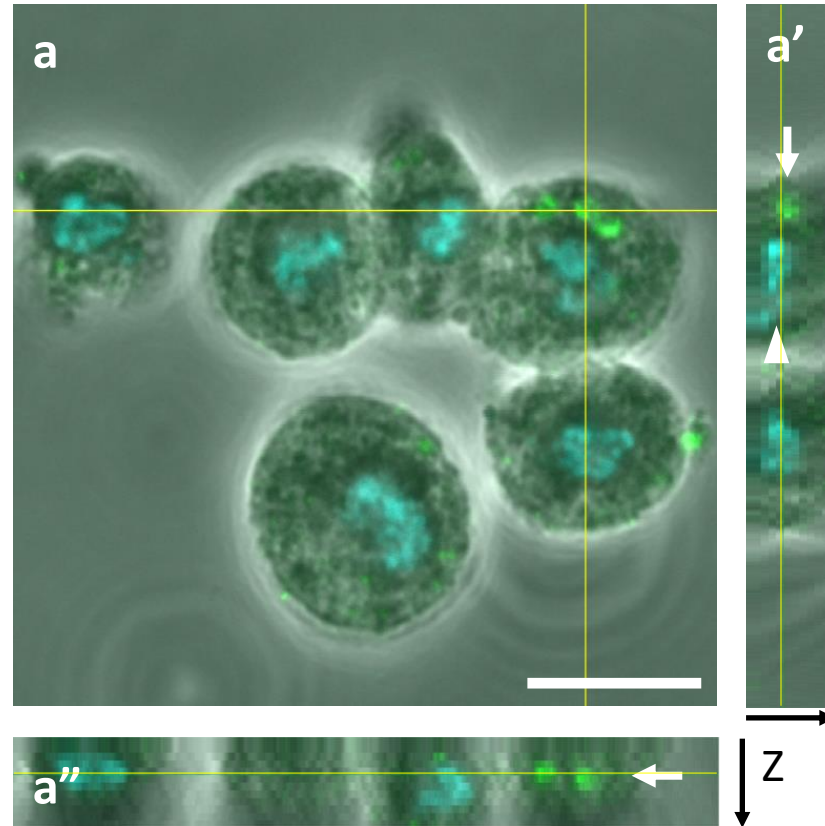

Figure S5

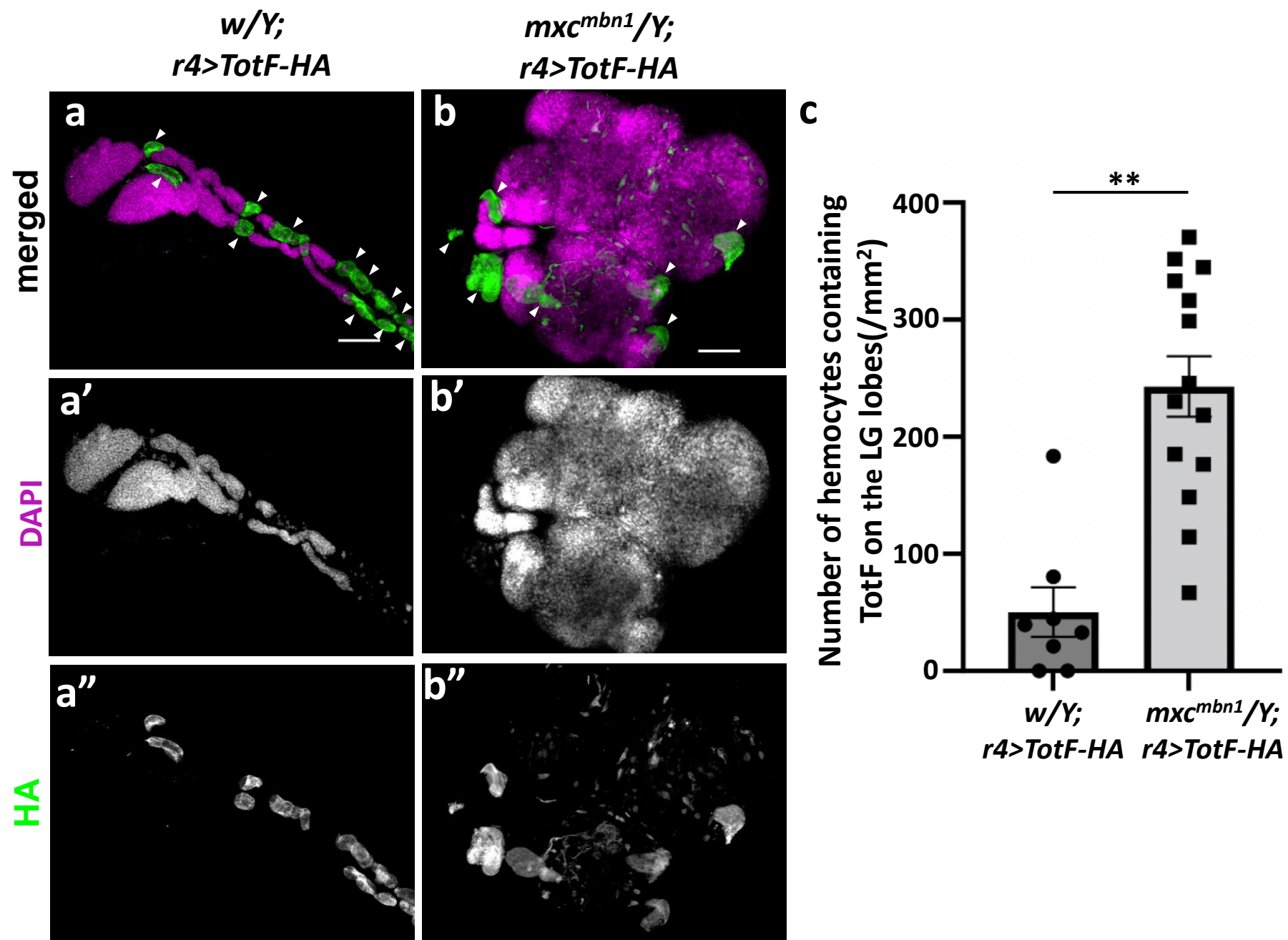

Supplement: Supplementary file 1 [file cells-12-02047-s001.zip › cells-2511802-supplementary.pdf]
